# Supplementary material for: Resolving the full spectrum of human genome variation using Linked-Reads
Source: Genome Res. 2019 Apr;29(4):635–45. doi: 10.1101/gr.234443.118 (PMC6442396; doi:10.1101/gr.234443.118)
Supplement: Supplemental Material [file supp_gr.234443.118_Supplemental_longranger-2.2.zip › longranger-2.2.tar.bz2!/lr2.2-public/lib/stan/cmdstan/stan/doc/api/html/structstan_1_1math_1_1coupled__ode__system.html]

Stan: stan::math::coupled\_ode\_system< F, T1, T2 > Struct Template Reference


|  |  |
| --- | --- |
|  | Stan  2.5.0  probability, sampling & optimization |


- Main Page
- Related Pages
- Namespaces
- Classes
- Files

- Class List
- Class Index
- Class Hierarchy
- Class Members

All Classes Namespaces Files Functions Variables Typedefs Enumerations Enumerator Friends Macros Pages

- stan
- math
- coupled\_ode\_system

stan::math::coupled\_ode\_system< F, T1, T2 > Struct Template Reference

Base template class for a coupled ordinary differential equation system, which adds sensitivities to the base system.
More...

`#include <coupled_ode_system.hpp>`

## Detailed Description

### template<typename F, typename T1, typename T2> struct stan::math::coupled\_ode\_system< F, T1, T2 >

Base template class for a coupled ordinary differential equation system, which adds sensitivities to the base system.

This template class declaration should not be instantiated directly — it is just here to serve as a base for its specializations, some of which are defined in namespace `stan::aggrad`.

Template Parameters
:   |  |  |
    | --- | --- |
    | F | the functor for the base ode system |
    | T1 | type of the initial state |
    | T2 | type of the parameters |

Definition at line 27 of file coupled\_ode\_system.hpp.

---

The documentation for this struct was generated from the following file:

- src/stan/math/ode/coupled\_ode\_system.hpp


---

[ Stan Home Page ]

*© 2011–2014,
Stan Development Team.*
